# When Fatigue Was Vasculitis: A Case of Limited PR3 ANCA-Positive Granulomatosis With Polyangiitis

**DOI:** 10.7759/cureus.109992

**Published:** 2026-05-31

**Authors:** Montasir Elmobark, Wisam Ali

**Affiliations:** 1 General Internal Medicine, University Hospital of North Tees, Durham, GBR; 2 Emergency, Best Care Hospital, Khartoum, SDN

**Keywords:** anca-associated vasculitis, chronic maxillary sinusitis, diagnostic delay, proteinase 3 (pr3)-positive granulomatosis with polyangiitis (gpa), secondary amenorrhea

## Abstract

Granulomatosis with polyangiitis (GPA) is a rare multisystem necrotizing vasculitis involving small to medium-sized vessels and is commonly associated with proteinase 3 antineutrophil cytoplasmic antibodies (PR3 ANCA). While pulmonary and renal manifestations are classically recognized, atypical presentations with predominant constitutional, sinonasal, ophthalmological, and endocrine manifestations may result in substantial diagnostic delay.

We present the case of a 28-year-old woman with a two-year history of profound fatigue, generalized myalgia, anosmia, chronic sinonasal symptoms, headaches, painful eye movements, and secondary amenorrhea. Her symptoms progressed to significant impairment in activities of daily living because of severe fatigue and lower limb pain. The patient underwent extensive hematological and endocrine investigations, including lymph node biopsy and pituitary hormonal assessment, before the eventual diagnosis of PR3 ANCA-positive GPA. Computed tomography imaging demonstrated complete opacification of the right maxillary sinus with additional ethmoidal mucosal thickening, while autoimmune serology demonstrated strongly positive anti-PR3 antibodies with negative anti-myeloperoxidase (MPO) antibodies. The patient subsequently demonstrated marked symptomatic improvement following the initiation of oral prednisolone therapy.

This case highlights the diagnostic challenges associated with ear, nose, and throat (ENT) predominant GPA in the absence of overt renal disease and emphasizes the importance of considering ANCA-associated vasculitis in patients with chronic inflammatory symptoms and refractory sinonasal disease.

## Introduction

Granulomatosis with polyangiitis (GPA) is an antineutrophil cytoplasmic antibody (ANCA)-associated vasculitis characterized by granulomatous inflammation and necrotizing vasculitis affecting predominantly the upper respiratory tract, lungs, and kidneys [1]. Although pulmonary renal syndrome represents the classical severe phenotype, the disease demonstrates substantial clinical heterogeneity and may present with localized or atypical manifestations resulting in delayed diagnosis [2].

Upper respiratory tract involvement is among the most common features of GPA and may include chronic sinusitis, anosmia, nasal crusting, epistaxis, and otologic manifestations [3]. Endocrine manifestations, however, remain uncommon and under-recognized. Pituitary involvement has been reported in isolated case reports and may occur through inflammatory involvement of the pituitary gland or stalk. It may manifest with amenorrhea, visual symptoms, headaches, and hormonal abnormalities mimicking pituitary adenoma or inflammatory hypophysitis [4,5].

We report a case of delayed diagnosis of proteinase 3 (PR3) ANCA-positive GPA presenting predominantly with constitutional, sinonasal, ophthalmological, and endocrine manifestations without significant renal impairment.

## Case presentation

A 28-year-old woman presented with a two-year history of progressive constitutional and multisystem symptoms, including profound fatigue, generalized myalgia, recurrent sinonasal symptoms, anosmia, headaches, painful eye movements, and diffuse body pain.

The patient described severe fatigue and exhaustion, significantly impairing her daily activities and functional status. She reported progressive limitation in routine activities because of generalized pain and lower limb discomfort. Standing from a seated position frequently precipitated significant bilateral leg pain and worsening fatigue.

During October 2025, the patient experienced a rapid, unintentional weight gain of approximately two stones despite poor appetite. This was followed by subsequent weight loss over the following months. In January 2026, she developed secondary amenorrhea, prompting endocrine evaluation.

Additional symptoms included intermittent loss of taste, intention tremor, diffuse abdominal discomfort, and transient episodes of right-sided facial and neck swelling associated with a non-pruritic facial macular rash. Persistent perioral and chin lesions with delayed healing were also reported.

The patient underwent hematological assessment following the identification of cervical lymphadenopathy on computed tomography imaging. Lymph node biopsy performed at an external hospital demonstrated reactive lymphadenitis with no evidence of malignancy, according to the available clinical documentation. The available external pathology documentation did not report malignancy; however, it did not specifically comment on the presence or absence of granulomatous inflammation or vasculitis. The original histopathology images were not available for inclusion. The patient was subsequently discharged from hematology follow-up.

Examination

On examination, the patient appeared fatigued but was hemodynamically stable. Chest auscultation was unremarkable. Abdominal examination demonstrated mild generalized tenderness without guarding or organomegaly. Musculoskeletal examination demonstrated diffuse joint tenderness without synovitis or joint swelling. No focal neurological deficit was identified.

Investigations

Laboratory investigations demonstrated persistently elevated inflammatory markers and positive PR3 ANCA serology (Table 1).

**Table 1:** Summary of laboratory investigations PR3 ANCA: proteinase 3 antineutrophil cytoplasmic antibodies; ANA: antinuclear antibody; MPO ANCA: myeloperoxidase antineutrophil cytoplasmic antibodies; ACTH: adrenocorticotropic hormone; IGF-1: insulin-like growth factor 1; CCP: cyclic citrullinated peptide; dsDNA: double stranded DNA; ENA: extractable nuclear antigen

Investigation	Result	Reference range
PR3 ANCA	32 IU/mL	Negative
MPO ANCA	0.28 IU/mL	<3.5 IU/mL
C-reactive protein	30-40 mg/L	<5 mg/L
Erythrocyte sedimentation rate	40 mm/hour	<12 mm/hour
White blood cell count	11.3×10⁹/L	4.0-11.0×10⁹/L
Neutrophils	8.6 ×10⁹/L	2.0-7.5×10⁹/L
Hemoglobin	126 g/L	115-165 g/L
Platelet count	316×10⁹/L	150-400×10⁹/L
Creatinine	57 µmol/L	49-90 µmol/L
Urea	4.2 mmol/L	2.5-7.8 mmol/L
Sodium	140 mmol/L	133-146 mmol/L
Potassium	4.1 mmol/L	3.5-5.3 mmol/L
Adjusted calcium	2.30 mmol/L	2.20-2.60 mmol/L
Serum osmolality	310 mOsm/kg	275-295 mOsm/kg
ACTH	6 ng/L	7.0-63.0 ng/L
IGF-1	38.5 nmol/L	10-38 nmol/L
ANA	Negative	Negative
Anti-dsDNA	Negative	Negative
ENA panel	Negative	Negative
Rheumatoid factor	Negative	Negative
Anti-CCP antibodies	Negative	Negative
Urinalysis	Trace proteinuria	Negative

The mildly elevated serum osmolality was considered in the context of the patient’s endocrine symptoms; however, it was not markedly elevated and was not supported by paired urine osmolality or urine output data to confirm central diabetes insipidus. In addition, the available pituitary hormonal assessment did not demonstrate convincing hypopituitarism. Therefore, endocrine involvement was considered possible but remained unconfirmed.

Computed tomography imaging of the head demonstrated complete opacification of the right maxillary antrum with mucosal thickening involving the left maxillary sinus and right ethmoid air cells. No intracranial hemorrhage, hydrocephalus, or pituitary macroadenoma was identified.

Representative computed tomography images demonstrating complete opacification of the right maxillary sinus and ethmoidal mucosal thickening are shown in Figure 1.

**Figure 1:**
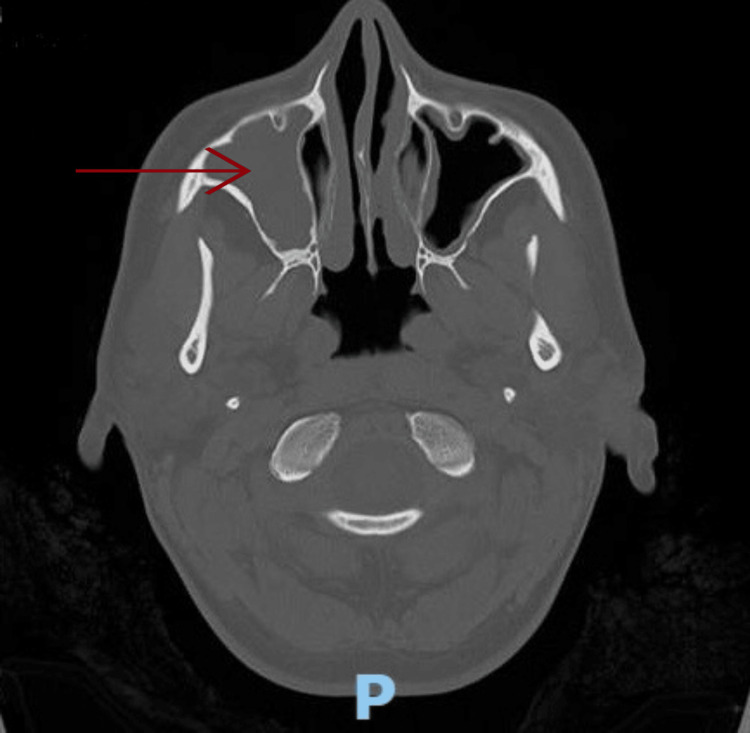
CT head Computed tomography image of the head demonstrating complete opacification of the right maxillary sinus with associated ethmoidal mucosal thickening (red arrow). CT: computed tomography

There was no radiological evidence of pulmonary involvement, and renal function remained preserved throughout assessment.

Diagnosis

Given the constellation of chronic sinonasal disease, anosmia, painful eye movements, constitutional inflammatory symptoms, elevated inflammatory markers, and positive PR3 ANCA serology, the overall clinical picture was considered highly suggestive of limited PR3 ANCA-associated GPA following rheumatology and dermatology assessment. Computed tomography imaging demonstrated significant sinonasal inflammatory disease involving the right maxillary sinus and ethmoid air cells.

Treatment and outcome

The patient was commenced on oral prednisolone 60 mg once daily with rapid symptomatic improvement. Shortly after treatment initiation, she reported marked improvement in fatigue, generalized pain, and constitutional symptoms, describing herself as feeling significantly better than at any point during the preceding two years. 

At the time of writing, the patient had not yet attended a specialist rheumatology follow-up. A steroid-sparing remission maintenance agent had therefore not yet been initiated. Longer-term immunosuppressive therapy, including consideration of rituximab, methotrexate, or azathioprine, was planned to be determined at follow-up according to disease activity, organ involvement, treatment response, and tolerability.

A chronological summary of the patient’s clinical course is presented in Table 2.

**Table 2:** Chronological summary of clinical events CT: computed tomography; PR3 ANCA: proteinase 3 antineutrophil cytoplasmic antibodies

Date	Clinical event
Approximately two years before diagnosis	Onset of progressive constitutional and multisystem symptoms, including profound fatigue, generalized myalgia, recurrent sinonasal symptoms, anosmia, headaches, painful eye movements, and diffuse body pain
October 2025	Rapid unintentional weight gain of approximately two stones despite poor appetite
Following months	Subsequent weight loss
January 2026	Development of secondary amenorrhea, prompting endocrine evaluation
January 2026	Cervical lymphadenopathy identified on computed tomography imaging
February 2026	External lymph node biopsy demonstrated reactive lymphadenitis with no evidence of malignancy, according to available clinical documentation
Later 2026	CT imaging demonstrated sinus opacification and ethmoidal thickening
Later 2026	Laboratory investigations demonstrated persistently elevated inflammatory markers and positive PR3 ANCA serology
April 2026	Overall clinical picture considered highly suggestive of limited PR3 ANCA-associated granulomatosis with polyangiitis; prednisolone therapy was commenced
Shortly after treatment	Marked symptomatic improvement reported, including improvement in fatigue, generalized pain, and constitutional symptoms

## Discussion

GPA is a rare multisystem autoimmune vasculitis characterized by necrotizing granulomatous inflammation and small vessel vasculitis [1]. Although pulmonary renal syndrome represents the classical severe phenotype, ear, nose, and throat (ENT) predominant or localized disease may lead to prolonged diagnostic delay because of non-specific manifestations and overlap with more common conditions [2].

This case demonstrates several clinically important learning points. First, chronic sinonasal symptoms associated with anosmia and systemic inflammatory manifestations should raise suspicion for systemic inflammatory disease rather than uncomplicated chronic sinusitis. ENT involvement occurs in the majority of patients with GPA and may precede systemic disease by several years [3].

Second, this case illustrates the diagnostic difficulty associated with GPA in the absence of overt renal involvement. Despite prolonged symptoms, the patient maintained normal renal function with only trace proteinuria. Such presentations may lead clinicians toward alternative diagnoses, including endocrine disease, occult malignancy, connective tissue disease, chronic infection, or neurological pathology.

Third, this case highlights the uncommon endocrine manifestations that may be considered in patients with GPA. Pituitary involvement remains rare but increasingly recognized in ANCA-associated vasculitis. Reported manifestations include amenorrhea, diabetes insipidus, headaches, visual symptoms, hypogonadism, and pituitary hormone abnormalities [4,5]. In this patient, secondary amenorrhea together with headaches, painful eye movements, mildly elevated serum osmolality, and mildly reduced ACTH initially prompted consideration of pituitary pathology. However, the serum osmolality was not markedly elevated and was not supported by paired urine studies to confirm central diabetes insipidus. The available endocrine assessment also did not demonstrate convincing hypopituitarism. Pituitary MRI was therefore considered but deferred once the diagnosis of PR3 ANCA-positive GPA was established on the basis of chronic sinonasal disease, systemic inflammatory symptoms, strongly positive PR3 ANCA serology, exclusion of alternative diagnoses, and subsequent clinical response to glucocorticoid therapy. Therefore, possible hypothalamic pituitary involvement was considered but remained unconfirmed.

Painful eye movements in GPA warrant careful attention because orbital inflammation, episcleritis, scleritis, and optic nerve involvement may occur and may threaten vision if untreated [6]. The coexistence of painful extraocular movements, chronic sinus disease, and strongly positive PR3 antibodies significantly strengthened clinical suspicion for GPA in this case.

The patient’s dramatic symptomatic response following corticosteroid therapy further supported the inflammatory vasculitic nature of the disease process. The marked improvement after two years of debilitating symptoms highlights the morbidity associated with delayed recognition of atypical GPA presentations.

Recent European Alliance of Associations for Rheumatology (EULAR) and British Society for Rheumatology recommendations emphasize early recognition of ANCA-associated vasculitis and support rituximab-based remission induction strategies in many patients with GPA [1,7].

## Conclusions

This case illustrates an atypical presentation of PR3 ANCA-positive GPA characterized predominantly by constitutional, sinonasal, ophthalmological, and possible endocrine manifestations without significant renal involvement. Persistent inflammatory symptoms associated with chronic sinonasal disease and anosmia should prompt consideration of ANCA-associated vasculitis, particularly when routine investigations fail to establish an alternative diagnosis. Early multidisciplinary assessment and recognition are essential to prevent prolonged morbidity and delayed treatment.
